# Training Process in Multidisciplinary Health Residencies with a View to Interprofessionalism

**DOI:** 10.1590/0034-7167-2025-0329

**Published:** 2026-11-06

**Authors:** Sâmara Fontes Fernandes, Rodrigo Jácob Moreira de Freitas, Fernando Jeferson Queiroz dos Santos, Márcio Danillo de Assis Santos, Lucas Souza Leite, Maria Rocineide Ferreira da Silva

**Affiliations:** I Universidade do Estado do Rio Grande do Norte. Mossoró, Rio Grande do Norte, Brazil; II Universidade do Estado do Rio Grande do Norte. Pau dos Ferros, Rio Grande do Norte, Brazil; III Universidade Estadual do Ceará. Fortaleza, Ceará, Brazil

**Keywords:** Professional Training in Health, Interprofessionality, Interdisciplinary Communication, Grounded Theory, Health Residency., Formación Profesional en Salud, Interprofesionalidad, Comunicación Interdisciplinaria, Teoría Fundamentada, Residencia de Salud.

## Abstract

**Objectives:**

to develop a theoretical model on the training process in Multidisciplinary Health Residencies.

**Methods:**

research grounded in Straussian Grounded Theory, conducted in residency programs in the countryside of Rio Grande do Norte, with 41 participants: students, faculty members, preceptors, and managers. Focus groups and interviews were conducted, guided by specific scripts. Data collection and analysis occurred simultaneously, using open, axial, and selective coding, culminating in the formulation of a paradigmatic model.

**Results:**

the theoretical model was structured into actions-interactions, conditions, and consequences, explaining the central category, “Understanding the interprofessional training process in Multidisciplinary Health Residencies”, which highlighted the relevance of preceptorship, integration strategies for collaborative work, pedagogical and organizational weaknesses, and the influence of management and healthcare services.

**Conclusions:**

the model explains the phenomenon in practice, highlighting the influence of social actors, strategies for consolidating interprofessional education, and strengthening collaborative work.

## RESUMEN

**Objetivos:**

elaborar un modelo teórico sobre el proceso formativo en las Residencias Multiprofesionales en Salud.

**Métodos:**

investigación fundamentada en la Teoría Fundamentada en los Datos de Strauss, realizada en programas de residencia en el interior de Rio Grande do Norte, con 41 participantes: estudiantes, docentes, preceptores y gestores. Se realizaron grupos focales y entrevistas, guiados por guiones específicos. La recolección y el análisis de los datos ocurrieron simultáneamente, utilizando codificación abierta, axial y selectiva, culminando en la formulación de un modelo paradigmático.

**Resultados:**

el modelo teórico se estructuró en acciones-interacciones, condiciones y consecuencias, explicando la categoría central, “Comprendiendo el proceso formativo interprofesional en las Residencias Multiprofesionales en Salud”, que evidenció la relevancia de la preceptoría, las estrategias de integración para el trabajo colaborativo, las fragilidades pedagógicas y organizativas y la influencia de la gestión y de los servicios de salud.

**Conclusiones:**

el modelo explica el fenómeno en la práctica, evidenciando la influencia de los actores sociales, las estrategias para la consolidación de la educación interprofesional y el fortalecimiento del trabajo colaborativo.

## INTRODUCTION

The population’s health needs have become progressively more complex due to demographic, epidemiological, and social changes, highlighting the limitations of a system still marked by fragmented care. This context reinforces the need to reorganize both services and training processes in order to prepare professionals capable of responding to the real demands of territories and working collaboratively^(1)^.

The reorientation of health education, driven by the principles of the Unified Health System (SUS - *Sistema Único de Saúde*), seeks to overcome models centered on biomedical and disciplinary logic, promoting critical and reflective professional profiles capable of working in integrated teams. This perspective values curricula aligned with the territory, participatory methodologies, and practices that encourage communication, cooperation, and shared responsibility among faculty, preceptors, and students^(2,3)^.

In this scenario, Multidisciplinary Health Residencies (MHRs) are configured as privileged spaces for in-service training, articulating teaching, practice, and local needs. These programs encourage collaborative work by bringing together professionals from different areas, contributing to comprehensive practices aligned with the principles of SUS. However, the administrative and pedagogical organization of residencies is still concentrated in institutions located in urban centers, which generates additional challenges for programs implemented in inland areas^(3,4)^.

Thus, in recent decades, public policies and interministerial actions in health and education have been created with the aim of reorienting training in the health field and reducing locoregional disparities. The Brazilian National Program for the Reorientation of Professional Training in Health (*Pró-Saúde*) stands out, as it encouraged the transformation of the training process through more effective integration between educational institutions and healthcare services, seeking to develop activities aligned with local realities^(3)^.

In the case of MHRs, there was a deconcentration of programs throughout the national territory rather than decentralization, since decisions regarding the entire training process-administrative, pedagogical, and financial-remained centralized in the hands of the proposing institutions, still located in large and medium-sized cities^(3)^.

The MHR proposal is innovative and interdisciplinary, including various healthcare professions to develop continuing education and articulate interdisciplinary knowledge across different areas, qualifying work, meeting the population’s health needs, and aligning with the principles and guidelines of SUS. The proposal is grounded in local and regional realities and, therefore, in territories’ priority needs^(4)^.

Thus, understanding the professional training developed by MHR in the territories, especially in inland cities, is a strategy to understand care models and health practices developed in that space, as well as to identify whether they respect the training principles that guide lato sensu professional education in health and whether they meet the population’s real health, social, and territorial needs. Therefore, the following question arises: how does the training process occur in MHR in inland cities of the state of Rio Grande do Norte (RN)?

Despite the expansion of MHRs, there is a scarcity of studies that build theoretical models capable of explaining how training occurs in these contexts, especially under the influence of inlandization, service organization, and collaboration among different professional categories.

## OBJECTIVES

To develop a theoretical model that explains these elements from the perspective of the various actors involved in the training process^(5,6)^.

## METHODS

### Ethical aspects

Authorization was requested from the selected MHRPs or from the institutions responsible for research in order to obtain local permission for data collection. The study was conducted in accordance with national and international ethical guidelines and was approved by the Research Ethics Committee of *Universidade Estadual do Ceará*, whose approval is attached to this submission.

### Study design

This is a descriptive study with a qualitative approach, conducted from the perspective of Grounded Theory (GT). In this case, the relativist or Straussian strand was adopted, in which the researcher is responsible for interpreting the data, using open, axial, and selective coding, as well as the paradigmatic model to organize the collected data^(6)^. Guided by its conceptual and theoretical principles, the study adopts a qualitative approach, seeking to understand the social phenomenon from the perspectives of different social actors involved in the process.

The conduct and reporting of this study followed COnsolidated criteria for REporting Qualitative research (COREQ) recommendations, an instrument composed of 32 items that guide transparency and rigor in the presentation of qualitative research, especially those using interviews and focus groups (FGs). The checklist was used as a guide for organizing methodological stages, describing data collection and analysis procedures, and ensuring greater clarity in the presentation of results.

### Researcher reflexivity

The principal researcher is a healthcare professional with previous experience in qualitative research and involvement in MHRs. She has a background in nursing and experience in training processes and health management, which facilitated understanding of the field but also required constant vigilance to minimize interpretative biases. Before data collection, the researcher had no direct relationship with participants, thus reducing potential asymmetrical influences. Throughout the process, memos and reflective journals were developed to make explicit positions, assumptions, and preliminary interpretations, ensuring greater transparency in the analytical process.

The interviews and FGs were conducted by a female researcher, PhD in health sciences, with academic and professional experience in qualitative research and in the field of multidisciplinary health education. At the time of the study, the researcher worked as a faculty member in a public higher education institution. Participants were informed about the study objectives, the researcher’s academic background, and the voluntary nature of participation.

### Research setting and sample definition

The research was conducted in four Multidisciplinary Health Residency Programs (MHRPs), located in inland areas of RN, linked to the *Universidade Federal do Rio Grande do Norte* (UFRN) and the *Universidade do Estado do Rio Grande do Norte* (UERN), in the municipalities of Mossoró, Santa Cruz, and Caicó. Initially, eight MHRPs were identified in the state, but those located outside the capital were selected in order to focus on the inlandization of health training.

The sample was constructed through theoretical sampling, being defined throughout data collection and analysis, as needs for deepening and refinement of analytical categories emerged. A total of 41 participants took part, divided into four groups: 30 students, six preceptors, four faculty members, and one manager from a Municipal Health Department. No refusals or withdrawals were recorded during recruitment and data collection.

Participants were intentionally selected, considering their direct link to the study object. Residents in the final year, preceptors, faculty members, and managers active in MHRPs in inland RN, aged 18 years or older, were included. Individuals on leave, first-year residents, and those linked to residencies in the capital were excluded. Data collection continued until theoretical saturation was reached.

Initially, eight MHRPs were identified across the state, covering different areas of practice and linked to UFRN and UERN, headquartered in four municipalities: Natal, Santa Cruz, Caicó, and Mossoró. However, aiming to investigate inlandization in *lato sensu* graduate health education within MHRPs, the study focused on programs located in inland cities of RN. Thus, the four MHRPs included were: MHRP in Family Health (UERN/Mossoró); MHRP in Maternal and Child Health (UFRN/Santa Cruz); MHRP in Primary Care (UFRN/Caicó); and MHRP in Maternal and Child Health (UFRN/Caicó).

Sample delimitation in research guided by GT was carried out through theoretical sampling. Therefore, the study sample was constructed throughout the data collection and analysis process, as information emerged indicating the need to gather more data to refine categories and develop theory. New information may be required during the process, and data collection may need to be redirected accordingly^(7)^.

### Data collection instrument

Data collection took place between November 2022 and August 2023. Initially, FGs were conducted with residents from the MHRPs, a technique that brings together six to 12 homogeneous participants to discuss different perspectives. Residents were invited by email, and meetings were held in private settings. One FG was planned for each MHRP; however, only two were conducted-one in Mossoró and another including residents from Caicó-reaching theoretical saturation.

The FG script included four guiding questions addressing the understanding of Interprofessional Education (IPE), the evaluation of the training process, the impact of MHRPs on healthcare services, and the competencies developed. After each FG, the data were transcribed, analyzed, and diagrammed, leading to new questions that required expansion of the sample.

New hypotheses led to the conduction of semi-structured interviews with preceptors, faculty members, and managers, also scheduled via email and carried out in private settings^(6)^.

In total, two FGs were conducted, each lasting an average of 90 minutes, held in reserved rooms of partner institutions, ensuring privacy and confidentiality. Seventeen individual semi-structured interviews were also conducted, with an average duration of 45 to 60 minutes, carried out in person in private spaces within healthcare services or academic institutions. All interviews were audio-recorded and fully transcribed by the researcher.

During the interviews and FGs, only the participants and the researcher were present. The sample consisted of residents, preceptors, faculty members, and one health manager, with different professional backgrounds and varying lengths of experience in Multidisciplinary Residency Programs.

For faculty members and preceptors, the script addressed their role in the residency, work organization, evaluation of interprofessional training, the impact of MHRPs, and the competencies developed. For managers, the questions focused on the role of management, articulation with training institutions, strategies for strengthening the MHRP, and impacts on the territory.

During the FGs and interviews, field notes were recorded, including observations related to the data collection environment, interactions among participants, nonverbal expressions, moments of agreement or tension in discourse, and the researcher’s preliminary analytical impressions. These notes were incorporated into the analytical process as complementary material, contributing to deeper data interpretation and strengthening qualitative rigor.

Each interview was transcribed and analyzed until theoretical saturation was reached, with no emergence of new properties or dimensions in the data.

No repeat interviews were conducted. The interview and FG transcripts were not returned individually to participants, as the adopted methodological strategy prioritized collective analytical validation of the emerging theoretical model. Instead of returning transcripts individually, a validation process of categories and the theoretical model was conducted with selected participants, allowing verification of interpretative coherence and strengthening the credibility of the findings.

Before data collection began, the scripts used in the FGs and semi-structured interviews underwent a pre-test with two professionals experienced in MHRPs who were not part of the final study sample. This procedure aimed to assess the clarity of the questions, the relevance of the topics addressed, and the average duration of application. After this stage, minor adjustments were made to the wording of some questions to enhance understanding and encourage more in-depth narratives from participants.

### Data processing and analysis

The analysis was carried out simultaneously with data collection, allowing validation of emerging concepts and hypotheses^(8)^. After data transcription, memos and diagrams were developed for the organization and storage of information, and were used throughout the entire research process.

The process of category construction occurred progressively and iteratively, according to the principles of GT. Initially, during open coding, the data were fragmented into units of meaning, generating initial codes derived directly from participants’ statements. Subsequently, in axial coding, these codes were compared, grouped, and reorganized into categories and subcategories based on their properties and dimensions. Finally, in selective coding, categories were integrated around a central phenomenon, allowing the construction of the theoretical model explaining the training process in MHRs.

The analysis followed three coding stages^(7)^:

- Open coding: identification of preliminary codes from raw data, forming initial categories and subcategories;
- Axial coding: regrouping and integration of categories and subcategories, explaining their properties, dimensions, and related phenomena^(8)^;
- Selective coding: construction of an explanatory theoretical scheme, with identification of the central category and formulation of a substantive theory or conceptual model^(9)^.

The paradigmatic model was used to structure the studied phenomenon, connecting phenomenon, actions-interactions, conditions, and consequences.

Coding and data analysis were performed by the principal researcher, following the stages of open, axial, and selective coding, with analytical rigor ensured through constant comparison and theoretical saturation.

Throughout this analytical process, theoretical memos and analytical diagrams were developed, which supported the visualization of relationships among categories, enhancing conceptual density and paradigmatic model construction.

The analysis did not use specific software, such as NVivo or Atlas.ti. Coding was conducted manually, strictly following the procedures proposed by Straussian GT. This choice was based on the understanding that manual processing favors greater immersion, theoretical sensitivity, and proximity of the researcher to the data, as recommended by classical GT authors.

To strengthen the credibility of the findings, validation was conducted with a group of four participants (two residents, one preceptor, and one faculty member), who assessed the coherence of the categories and the theoretical model. Their contributions were incorporated into the final coding process, without the need for structural adjustments, confirming the analytical consistency.

## RESULTS

During the data analysis process, the data were organized into three components of the theoretical model-actions-interactions, conditions, and consequences-as shown in Chart 1. From the relationships between the data, categories and subcategories emerge, each containing its own elements that describe and explain the phenomenon under study. Thus, the central category of the phenomenon in this study was entitled “Understanding the interprofessional training process in Multidisciplinary Health Residencies”.

**Chart 1:** Organization of the paradigmatic model regarding the training process of Multidisciplinary Health Residencies, Mossoró, Rio Grande do Norte, Brazil, 2023

	CATEGORIES	SUBCATEGORIES
**CENTRAL CATEGORY**	Understanding the interprofessional training process in Multidisciplinary Health Residencies	-
**ACTIONS-INTERACTIONS**	Influence of preceptorship on the training process	- Performance of core and field preceptors are important instruments for consolidating interprofessional work. - Absence of field preceptors from all disciplinary areas is an obstacle in the training process. - There is a lack of uniformity in the payment of scholarships as an incentive for participation in Multidisciplinary Health Residencies. - Absence of a continuous qualification process for preceptors.
	Strategies for consolidating interprofessional work	- Shared care across all disciplines encourages interprofessional teamwork. - Coordination between different residency programs, including medical residencies, strengthens interinstitutional collaboration. - Lack of uniformity in systematic team meetings to refine work processes. - Uniprofessional care is necessary for the disciplinary work process and the development of competencies. - Family Health Support Center disarticulation compromised the training process of Multidisciplinary Health Residencies focused on Primary Care.
	Needs involved in the training process in Multidisciplinary Health Residencies	- Presence of various internship sites encourages teamwork and the development of different skills. - Shared responsibility of municipal administrations for Multidisciplinary Health Residencies is necessary to strengthen them in the territory. - Need to strengthen the understanding of user-centered care and health needs as the guiding principle of health practices. - Encouragement for faculty members and preceptors to participate in Multidisciplinary Health Residencies.
**CONDITIONS**	Influence of undergraduate education on the training process in residency	- Lack of sufficient interdisciplinary experiences and discussions during undergraduate studies. - Fragmented, disciplinary training guided by a biological logic. - Difficulty in developing teamwork between different disciplines, even during undergraduate studies.
	Pedagogical organization of Multidisciplinary Health Residencies	- Organized training over two years with theoretical and practical components. - Presence of diverse social facilities as internship sites. - Presence of principles of interprofessional education. - Weakened disposition in the academic organization and relationship between the social actors involved (faculty members, preceptors, students, and managers). - Difficulty in the articulation and training of preceptors.
	Influence of healthcare service organization on training	- Fragmented, segregated, and predominantly disciplinary work processes. - Weakness in teamwork. - Lack of qualifications for healthcare professionals in the services. - The role of field and core preceptorship as a key instrument for organizing the work process.
	Role of municipal management as the executing institution	- Collaboration between implementing and training institutions. - Financial organization to provide scholarships and structure healthcare services. - Need for on-the-job training to change the professional and epidemiological profile of the municipality.
**CONSEQUENCES**	Development of competencies in Multidisciplinary Health Residencies	- Understanding the development of common, transversal, and collaborative competencies. - Development of collaborative practice. - Development of interprofessional teamwork. - Development of the centrality of care, health needs, and interpersonal communication. - Difficulties in consolidating interprofessional education.
	Impact of Multidisciplinary Health Residencies in the territories	- Presence of residential care in healthcare services improves healthcare quality. - Development of more comprehensive care. - Development of new flows and specialized care services. - Creation of bonds with the population, improving their quality of life. - Increased effectiveness of healthcare services. - Development and refinement of work processes. - Transformation of municipal health indicators.

The “actions-interactions” component corresponds to the responses expressed to events or problematic situations. Within this component, statements emerged that supported three categories and their respective subcategories. The first category, “Influence of preceptorship on the training process”, identified that the role of preceptors is essential and represents one of the structuring axes of this process in residencies, as these professionals act as mediators between academia and healthcare services, in addition to guiding students through the work processes established in training settings. Therefore, the selection of preceptors must be rigorous, as they need to be committed and qualified for the training process due to their direct influence on in-service training and on the development of competencies and skills. This is illustrated in the following statement:

[...] *field preceptorship consists of preceptors who are responsible for a team within a specific service. The team is composed of all residents from all categories. Here, we work with a base service and a support service, and they are fixed to one service. There is also a core preceptor, who is responsible for each category-all residents of the same profession-for weekly meetings to address any gaps a resident may have or to expand discussions about activities.* (Student 4)

In the second category, “Strategies for consolidating interprofessional work”, the statements revealed essential instruments developed within residencies that support the consolidation of interprofessional work. Shared care among different disciplines in training settings and articulation among different residencies, including medical residencies, are the main strategies used to encourage interdisciplinary teamwork. However, the need to strengthen disciplinary competencies through individual work processes and uniprofessional care was also identified, as a way to support the development of collaborative competencies and interprofessional practice. This is evidenced in the following statements:

*Today, we are making a move to integrate residency programs, including medical residencies. In other words, the interprofessional learning seminar component is part of Primary Care, but it also encompasses maternal and child health and family and community medicine* [...] *and so this case discussion, which previously only happened in Primary Care, now happens with everyone.* (Faculty member 3)

On the other hand, it is also stated that:

*Interprofessionalism implies sharing objectives* [...] *it’s about planning together and executing them. It doesn’t mean we eliminate single-professional practice; quite the contrary, it’s very necessary. And there was a conceptual confusion where residents were doing shared care, educational sidewalk sessions, operational groups, groups for pregnant women, and refusing to do individualized activity, that is, single-professional care, and that cannot happen.* (Faculty member 1)

In the third category, “Needs involved in the training process in Multidisciplinary Health Residencies”, it was highlighted that the diversity of training settings at different levels of complexity within the Health Care Network enriches the training process and contributes to the development of various competencies. However, greater commitment from municipal management is needed in this organization, as well as greater encouragement for faculty members and preceptors to participate in MHR, given the difficulty in assembling these teams. This directly influences the need to strengthen the understanding of user-centered care and health needs as guiding axes of health practices, as reinforced by the following statement:

*Today, we have the LGBT clinic, the street clinic, the women’s hospital* [...] *they pay for those hours that aren’t at the BHU, in those other locations, because, in reality, it’s not even like a shift; they would have to pay for eight hours a week, which is out of 48; they can only pay for 40, so they pay for shifts.* (Preceptor 5)

Despite the convergence regarding the relevance of interprofessionality as a structuring axis of training in MHRs, the findings revealed important variations and tensions among programs, practice settings, and professional categories. While some participants reported training experiences marked by strong integration among residents, preceptors, and services, others highlighted weaknesses in interprofessional articulation, especially in contexts where fragmented work processes prevail or where there is low adherence of service professionals to residency proposals.

Divergent perceptions were also observed among professional categories regarding the balance between shared practices and uniprofessional care, revealing conceptual and operational tensions concerning the role of interprofessionality in everyday training. These differences reflect not only institutional and territorial specificities but also previous training trajectories and distinct organizational conditions, reinforcing the complexity of the phenomenon under investigation and the need to understand interprofessional training as a heterogeneous, relational, and contextually situated process, as recommended by the analytical rigor criteria of COREQ-EQUATOR.

The “conditions” component addresses the reasons given by participants for the occurrence of events, as well as explanations for why they respond in a certain way to an action. Within this component, statements emerged that supported four categories and their respective subcategories. The first category, “Influence of undergraduate education on the training process in residency”, shows that the absence of discussions on IPE and interdisciplinary experiences in undergraduate programs weakens teamwork and makes the residency training process more difficult from an interprofessional perspective. This is illustrated by the following statement:

*Today, I see this especially because of the close collaboration between the three residency programs. Our biggest challenge is how the resident arrives here, because, mainly-and this isn’t meant to be negative-some undergraduate programs don’t include discussions about the SUS or interdisciplinary experience. They’re very used to staying in their little box, only teaching and not working with other professions.* (Faculty member 2)

In the second category, “Pedagogical organization of Multidisciplinary Health Residencies”, it was shown how these residencies are pedagogically structured in terms of workload, modules, and activities. The presence of IPE principles in the programs’ pedagogical structure was highlighted. However, significant difficulty was identified in articulating the social actors involved in meetings, projects, and discourse. This is illustrated in the following statement:

*It’s a multi-professional residency program. As it was originally conceived, it doesn’t address interprofessional education. However, we chose to include interprofessional education as one of the formative principles, right? And so we have one, or rather two, curricular components, but we have one called “seminars on interprofessional learning and interprofessional work”, so we have two curricular components that address this discussion.* (Faculty member 1)

In the third category, “Influence of healthcare service organization on training”, the predominance of a hospital-centered and biologicist logic in healthcare services was identified as a barrier to interdisciplinary teamwork, as it encourages fragmented and disciplinary professional practices. Thus, the need for continuous qualification within healthcare services is emphasized, as well as the selection of strategic preceptors to act in these settings and serve as instruments for transforming reality. This is illustrated below:

*I believe the difference in residency is seeing it in practice, because we still hear very little about interprofessionalism in undergraduate studies; it’s something you only hear about, you don’t experience it. I think it’s a huge advantage for us to be immersed in this reality. The vast majority is practical; we discover what interprofessionalism is within interprofessional care. But this teamwork is very difficult in practice. We have difficulties with some professionals in the services: the more senior ones.* (Student 10)

In the fourth category, “Role of municipal management as the executing institution”, it was found that although management recognizes the need for in-service training to change the professional and epidemiological profile of the municipality, it still acts in a limited and weakly articulated manner with the training institution, which weakens the training process. This is reinforced by the following statement:

*The role of management* [...] *it’s fundamental in supporting these movements. Management today understands the importance of the residency program; it values and supports it, and makes a significant effort to help execute the strategic activities of the residency program, but it still needs to coordinate better with us.* (Faculty member 4)

Finally, in the “consequences” component, which refers to expected or actual outcomes, two categories and their respective subcategories emerged. In the first category, “Development of competencies in Multidisciplinary Health Residencies”, it was observed that, despite difficulties in the training process, there is indeed the development of common, transversal, and collaborative competencies among students. It was also found that interpersonal communication is strengthened during the process and that it is possible to develop collaborative practice and interprofessional teamwork focused on the individual’s health needs and centered on the patient. This idea is reinforced in the following statements:

*The competencies we intentionally work on, that is, there is a clear intentionality; the main one is user-centeredness. It’s about the users’ health needs, centrality, interprofessional work, communication. We understand that centrality is what gives focus to the integration of interprofessional articulations, it’s not because of that* [...]. (Faculty member 2)

*Interprofessional education is not just bringing people, students and different professionals together in the same space; it’s much more complex than that; it’s much more than a meeting; it requires availability, so it’s not as simple as we imagine. It cannot be trivialized, and if we do, we will see later that we will not see the effects of interprofessional education. The problem is not interprofessional education, the problem is that we have never done interprofessional education* (Faculty member 3)

In the second category, “Impact of Multidisciplinary Health Residencies in the territories”, it was shown that the presence of residencies in healthcare services improves the quality of care, increases service resolvability, improves municipal health indicators, promotes more comprehensive care, refines work processes, creates new care pathways, and enhances articulation between levels of care. In addition, it positively impacts the quality of life of the individuals assisted. The location of MHRs in inland areas was not widely discussed nor considered highly relevant in the statements, suggesting that their impact is independent of location. This is reinforced by the following statement:

*Looking at the service I provide today, as a whole, I think our impact is much greater on community life* [...] *the actions we are able to provide to this population when I can meet with an interdisciplinary team, with comprehensive care* [...] *this is only done in services that have residents, so we have an impact on the lives of the families where we provide care. For me, that’s already the great impact of our existence* [...] *bringing quality of life to those who, in their social and economic context, would never be able to achieve it otherwise* [...]. (Student 15)

To present the theoretical model, an illustrative figure was developed regarding the interprofessional training process in MHRs, as shown in Figure 1.

**Figure 1:**
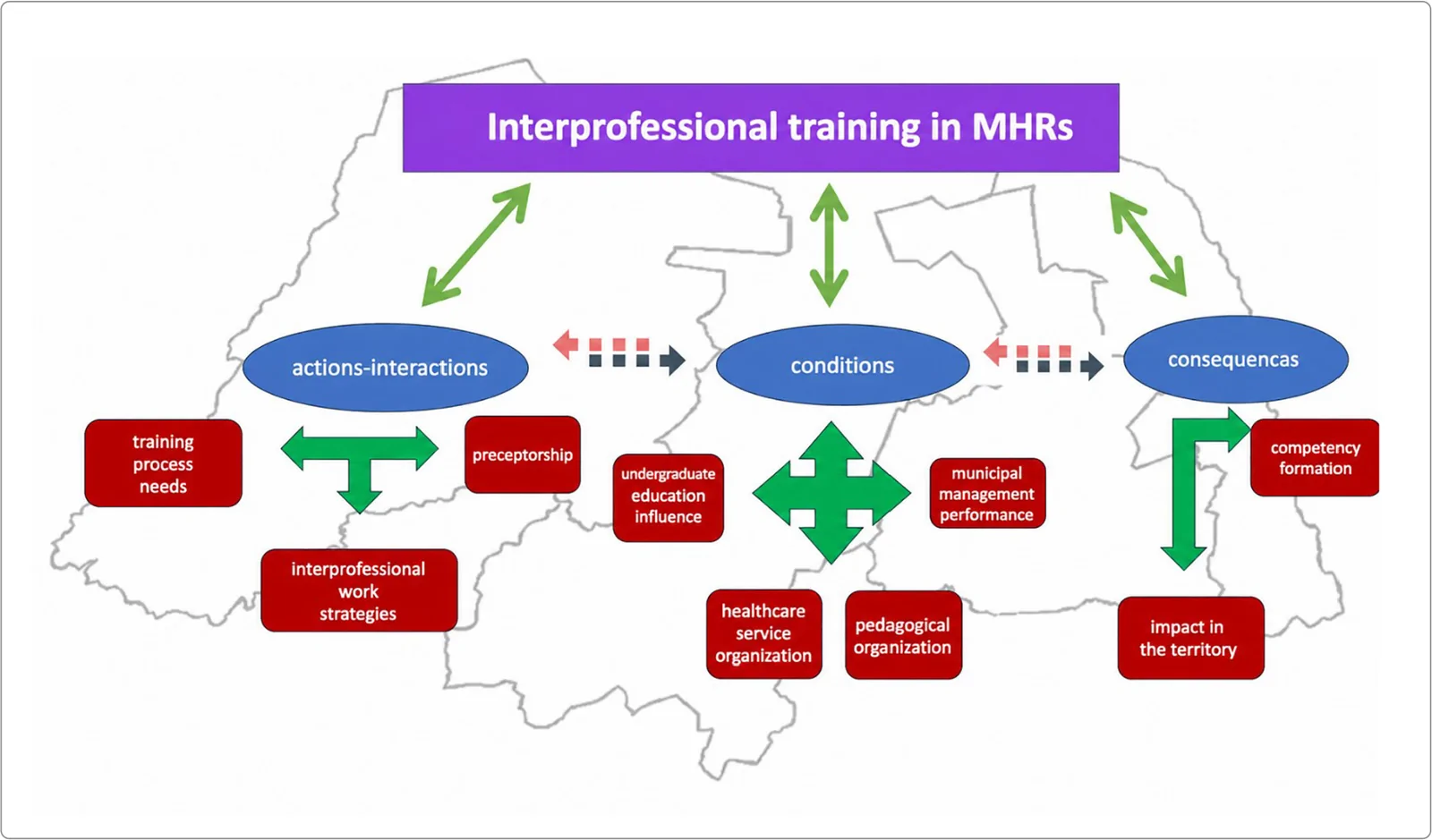
Diagram of the theoretical model regarding interprofessional training in Multidisciplinary Health Residencies, Mossoró, Rio Grande Norte, Brazil, 2023

In this model, the theoretical construct of the categories that emerged from participants’ statements synthesizes the main elements of the studied phenomenon. As observed, the concepts discussed made it possible to identify processes, practices, and challenges involved in interprofessional training in MHRs.

## DISCUSSION

These results converge with studies that highlight the central role of collaborative practices in improving care and strengthening communication among professionals. Studies show that integrated learning environments favor the development of common and collaborative competencies, which are essential for teamwork and comprehensive care^(10,11)^.

This shift moves health education away from a uniprofessional, biomedical, fragmented, and specialized model toward an interdisciplinary and interprofessional approach, grounded in teamwork, comprehensive care, and collaboration among healthcare professionals^(12,13)^.

Moreover, it promotes broader social awareness by addressing complex demands through innovative and collaborative practices, strengthening comprehensive, person-centered care, and supporting education and work in health from an interprofessional perspective^(14)^. It also contributes to the development of competencies and skills among students, such as negotiation, mediation, tolerance, dynamism, flexibility, autonomy, leadership, initiative, empathy, and dialogue, while encouraging shared practices and clarifying the roles and importance of each professional category within the healthcare team^(14)^.

Thus, in-service training developed by MHR creates a privileged space for interdisciplinary practice in real work settings, with potential for developing new care technologies and pedagogical practices such as IPE, especially when considering regional aspects and territorial needs, guiding education committed to social responsibility and interprofessional teamwork^(10)^.

IPE is considered a teaching-learning strategy committed to preparing professionals for interprofessional collaboration and teamwork. It is intersectorally articulated to address the growing complexity of population health needs, focusing on comprehensiveness and consequently impacting improvements in education, work dynamics, and health outcomes^(15)^.

IPE is characterized by the opportunity for two or more professions to learn together in an integrated manner, developing skills for teamwork and professional collaboration toward a common goal. It is based on interprofessional learning through methods that encourage interaction and the sharing of knowledge and responsibilities, enabling teamwork processes and collaboration among professionals^(10)^.

Despite these advances, structural weaknesses persist, such as limited articulation between municipal management and training institutions, difficulties in service work processes, and limitations stemming from prior training of professionals, often marked by disciplinary fragmentation. These elements directly influence the consolidation of collaborative work and the effectiveness of training experiences^(6,12)^.

Therefore, the interprofessional learning environment in health training optimizes problem-solving, facilitates teamwork, reinforces each professional’s role, promotes positive attitudes in the workplace, improves communication among professionals, and enhances patient care^(16)^.

Thus, IPE develops a teaching-learning process planned in an interprofessional manner based on a system of specific, common, and collaborative competencies^(17)^. These competencies strengthen the training of professionals capable of interprofessional teamwork while respecting professional identities and existing boundaries^(18)^.

Consequently, the training process based on IPE should be directed toward improving individual and collective skills to develop patient-centered care. This process should be grounded in learning experiences that promote group dynamics, case discussions, construction of Individualized Therapeutic Projects, among others, aiming to strengthen communication, trust, and teamwork. It is essential that participants discuss ideas, define core objectives and tasks, make shared decisions, assume responsibilities, and manage conflicts^(12)^.

It is important to highlight the relevance of shared care or interconsultation, recognized as an interdisciplinary activity among different professional categories, aiming at more comprehensive, patient-centered, and resolutive care. In this context, interdisciplinary knowledge is shared, fostering interprofessional practice^(19)^.

However, individual care and uniprofessional practice should not be neglected during the process, as it is necessary to develop specific competencies, which are enabled through individual and disciplinary work processes. Therefore, interprofessionality does not disregard disciplinary practice; rather, it recognizes that different competencies can be developed through both approaches^(20)^.

Moreover, MHRPs are capable of transforming healthcare services due to the close integration among faculty, preceptors, and residents, who are willing to critically reflect and act upon reality^(5)^. From this perspective, the positive impact of the preceptor’s role in residency programs is emphasized. The preceptor is responsible for directly supervising residents’ practical activities, connecting them to healthcare services and guiding their practices, thereby contributing to changes aligned with public policies and comprehensive care^(21)^.

However, preceptors must have minimum knowledge related to the teaching-learning process, curriculum, objectives, and values of the residency program in order to adequately perform their role, recognize the proposed competency model, and strengthen integration among teaching, service, and community^(21)^.

Municipal management is also one of the social actors involved in the training process of MHRs. Although it recognizes the potential of residencies in improving healthcare services and participates in planning actions-especially in structuring care networks-its involvement is still insufficient. Most studies show a lack of local management strategies to strengthen residencies, which are incorporated into routine service functioning without major adaptations to the teaching-learning process^(22)^.

On the other hand, financial incentives such as scholarships for students, faculty, and preceptors, as well as continuing education programs and hiring of residency graduates, appear as important initiatives for strengthening residencies in the territories. However, these are not universally established measures in MHR regulations, and therefore not all programs adopt them^(22)^.

The potential of MHRs is evident and widely recognized. For managers, the presence of residencies expands access to services, strengthens bonds, increases quality of care, and improves satisfaction with care provided to the population^(22)^. Graduates, in turn, recognize the impact of this training process on their professional practice, feeling more qualified and effective, contributing to health system resolvability and population quality of life^(23)^.

Another important element is clinical management developed by residents, which is guided by territorial demands, considers regional heterogeneity, monitors continuous care, stratifies risks, and organizes care pathways. This particularly impacts medicine, which traditionally focuses on curative care but, when integrated with multidisciplinary residencies, tends to adopt a health promotion paradigm centered on the individual, facilitating teamwork and professional collaboration^(23)^.

Therefore, the presence of MHRs in territories can restructure services, implement new actions and care pathways, mobilize continuing education, qualify professionals and care, positively impacting care management and strengthening territorial interventions. The findings of this study align with international perspectives on interprofessionality described by Reeves et al. and Barr et al., particularly regarding the need for institutional structures that support collaborative practices and participatory learning environments. Similarly, principles such as user-centered care, communication, and collaboration-widely discussed in the WHO Framework-also emerged as structural foundations in the training developed by the MHRs investigated^(24-26)^.

Thus, strengthening training in MHR requires strategies that qualify preceptors, institutionalize collective discussion spaces, and consolidate collaborative practices in daily service routines. These investments are essential to sustain a training model that responds to territorial needs and consolidates interprofessionality as a structuring axis of health practice^(10,18)^.

### Study limitations

The present study has as its main limitation the fact that it was conducted in only one federative state, specifically in residencies located in inland areas. This delimitation allows for an understanding of the local reality; however, it limits the generalization of results to other contexts and to regional and national levels. Additionally, the absence of first-year residents and the non-inclusion of all programs existing in the state may have hindered a more comprehensive analysis of the state context.

### Contributions to health

The study makes a significant contribution to the field of public health by providing a theoretical model that explains the interprofessional training process in MHRs, considering the perspective of different social actors involved. Thus, it can support pedagogical actions, continuing education strategies, and public policies aimed at improving residency programs. It also enables the strengthening of IPE as an essential training guideline, with the potential to transform professional practices and the organization of healthcare services.

## CONCLUSIONS

It is concluded that the theoretical model developed regarding the interprofessional training process in MHRs is capable of explaining how this phenomenon occurs in practice, considering the influence of all social actors involved, the strategies necessary to consolidate interprofessional education and work, as well as the social structures required to develop in-service training, also highlighting the difficulties throughout the process.

Therefore, the theoretical model guides interprofessional health training through strengthening IPE and raising awareness about the need to restructure the pedagogical and structural processes currently developed in MHRs. It is also proposed as a basis for interprofessional practice in health.

It is expected that this study contributes to health disciplines and nursing science by addressing knowledge gaps related to GT and enhancing scientific research and discussions on interprofessionality and interprofessional health training. The study is limited to the state of RN.

## Data Availability

The research data are available within the article.
