# Supplementary material for: Training Process in Multidisciplinary Health Residencies with a View to Interprofessionalism
Source: Rev Bras Enferm. 2026 Nov 6;79:e20250329. doi: 10.1590/0034-7167-2025-0329 (PMC13742974; doi:10.1590/0034-7167-2025-0329)
Supplement: Translation

# Translation: Processo Formativo nas Residências Multiprofissionais em Saúde com Vistas à Interprofissionalidade

**DOI:** 10.1590/0034-7167-2025-0329pt

**Authors:** Sâmara Fontes Fernandes, Rodrigo Jácob Moreira de Freitas, Fernando Jeferson Queiroz dos Santos, Márcio Danillo de Assis Santos, Lucas Souza Leite, Maria Rocineide Ferreira da Silva

**Affiliations:** I Universidade do Estado do Rio Grande do Norte. Mossoró, Rio Grande do Norte, Brasil; II Universidade do Estado do Rio Grande do Norte. Pau dos Ferros, Rio Grande do Norte, Brasil; III Universidade Estadual do Ceará. Fortaleza, Ceará, Brasil

**Keywords:** Formação Profissional em Saúde, Interprofissionalidade, Comunicação Interdisciplinar, Teoria Fundamentada, Residência em Saúde.

## Abstract

**Objetivos:**

elaborar modelo teórico sobre o processo formativo nas Residências Multiprofissionais em Saúde.

**Métodos:**

pesquisa fundamentada na Teoria Fundamentada nos Dados straussiana, realizada em programas de residência no interior do Rio Grande do Norte, com 41 participantes: discentes, docentes, preceptores e gestores. Foram realizados grupos focais e entrevistas, guiados por roteiros específicos. A coleta e análise de dados ocorreram simultaneamente, utilizando codificação aberta, axial e seletiva, culminando na formulação de modelo paradigmático.

**Resultados:**

o modelo teórico foi estruturado em ações-interações, condições e consequências, explicando a categoria central, “Compreendendo o processo formativo interprofissional nas Residências Multiprofissionais em Saúde”, que evidenciou a relevância da preceptoria, estratégias de integração para o trabalho colaborativo, fragilidades pedagógicas e organizacionais e a influência da gestão e dos serviços de saúde.

**Conclusões:**

o modelo explica o fenômeno na prática, evidenciando a influência dos atores sociais, as estratégias para consolidação da educação interprofissional e fortalecimento do trabalho colaborativo.

## INTRODUÇÃO

As necessidades de saúde da população tornaram-se progressivamente mais complexas em função das mudanças demográficas, epidemiológicas e sociais, evidenciando limitações de um sistema ainda marcado pela fragmentação do cuidado. Esse contexto reforça a exigência de reorganizar tanto os serviços quanto os processos formativos, de modo a preparar profissionais capazes de responder às demandas reais dos territórios e atuar em colaboração^(1)^.

A reorientação da formação em saúde, impulsionada pelos princípios do Sistema Único de Saúde (SUS), busca superar modelos centrados na lógica biomédica e disciplinar, promovendo perfis profissionais críticos, reflexivos e capazes de atuar em equipes de maneira integrada. Essa perspectiva valoriza currículos articulados ao território, metodologias participativas e práticas que estimulem a comunicação, a cooperação e a corresponsabilidade entre docentes, preceptores e discentes^(2,3)^.

Nesse cenário, as Residências Multiprofissionais em Saúde (RMSs) configuram-se como espaços privilegiados de formação em serviço, articulando ensino, prática e necessidades locais. Esses programas estimulam o trabalho colaborativo ao reunir profissionais de diferentes áreas, contribuindo para práticas integrais e alinhadas aos princípios do SUS. Entretanto, a organização administrativa e pedagógica das residências ainda se concentra em instituições situadas em centros urbanos, o que gera desafios adicionais para programas interiorizados^(3,4)^.

Assim, nas últimas décadas, políticas públicas e ações interministeriais de saúde e educação foram criadas com o objetivo de reorientar a formação na área da saúde e diminuir as disparidades locorregionais. Destaca-se o Programa Nacional de Reorientação da Formação Profissional em Saúde (Pró-Saúde), que incentivou a transformação do processo de formação a partir da integração mais efetiva entre instituições de ensino e serviços de saúde, buscando desenvolver atividades articuladas à realidade local^(3)^.

No caso das RMSs, ocorreu uma desconcentração dos programas em todo o território nacional ao invés de descentralização, visto que as decisões de todo o processo formativo, de maneira administrativa, pedagógica e financeira, ficaram centralizadas nas mãos das instituições proponentes, ainda localizadas nas cidades grandes e médias^(3)^.

A proposta da RMS é inovadora e interdisciplinar, incluindo diversas profissões da saúde para desenvolver educação permanente e articular saberes interdisciplinares entre diferentes áreas, qualificando o trabalho, atendendo às necessidades de saúde da população e aos princípios e diretrizes do SUS. A proposta se alicerça nas realidades locais e regionais e, portanto, nas necessidades prioritárias dos territórios^(4)^.

Desse modo, compreender a formação profissional desenvolvida pelas RMSs nos territórios, principalmente nas cidades interioranas, é uma estratégia para compreender os modelos de atenção e as práticas de saúde desenvolvidas naquele espaço, além de identificar se as mesmas respeitam os princípios formativos que guiam a formação profissional *lato sensu* em saúde e se atendem às reais necessidades de saúde, sociais e territoriais da população. Portanto, questiona-se: como ocorre o processo formativo nas RMSs nas cidades do interior do estado do Rio Grande do Norte (RN)?

Apesar da expansão das RMSs, observa-se escassez de estudos que construam modelos teóricos capazes de explicar como ocorre a formação nesses contextos, especialmente sob a influência da interiorização, da organização dos serviços e da colaboração entre diferentes categorias profissionais.

## OBJETIVOS

Elaborar um modelo teórico que explicite esses elementos a partir da perspectiva dos diversos atores envolvidos no processo formativo^(5,6)^.

## MÉTODOS

### Aspectos éticos

Solicitou-se a anuência junto aos PRMS elencados no estudo ou com as instâncias responsáveis pela pesquisa nas instituições, a fim de se obter permissão local para a realização da coleta de dados. O estudo foi conduzido conforme diretrizes éticas nacionais e internacionais e aprovado pelo Comitê de Ética em Pesquisa da Universidade Estadual do Ceará, cujo parecer está anexado à presente submissão.

### Tipo de estudo

Trata-se de estudo descritivo, com abordagem qualitativa, realizado na perspectiva da Teoria Fundamentada nos Dados (TFD). Nesse caso, será adotada a vertente relativista ou straussiana, na qual o pesquisador é responsável por interpretar os dados, utilizando o processo de codificação aberta, axial e seletiva, assim como o modelo paradigmático para organizar os dados coletados^(6)^. Guiando-se pelos princípios conceituais e teóricos da mesma, possui abordagem qualitativa, buscando compreender o fenômeno social sob as visões dos diferentes atores sociais envolvidos no processo.

A condução e a descrição deste estudo seguiram as recomendações do *COnsolidated Criteria for REporting Qualitative research* (COREQ), instrumento composto por 32 itens que orienta a transparência e o rigor na apresentação de pesquisas qualitativas, especialmente aquelas que utilizam entrevistas e grupos focais (GFs). O *checklist* foi utilizado como guia para a organização das etapas metodológicas, para a descrição dos procedimentos de coleta e de análise de dados e para garantir maior clareza na apresentação dos resultados.

### Reflexividade do pesquisador

A pesquisadora principal é profissional da área da saúde com experiência prévia em pesquisa qualitativa e atuação em RMSs. Possui formação em enfermagem e experiência em processos formativos e gestão em saúde, o que favoreceu a compreensão do campo, mas também exigiu vigilância constante para minimizar vieses interpretativos. Antes da coleta, a pesquisadora não possuía vínculo direto com os participantes, reduzindo, assim, possíveis influências assimétricas. Durante todo o processo, foram elaborados memorandos e diários reflexivos para explicitar posições, pressupostos e interpretações preliminares, garantindo maior transparência ao percurso analítico.

As entrevistas e os GFs foram conduzidos por pesquisadora do sexo feminino, doutora em ciências da saúde, com experiência acadêmica e profissional em pesquisa qualitativa e no campo da formação multiprofissional em saúde. À época do estudo, a pesquisadora atuava como docente em instituição pública de ensino superior. Os participantes foram informados sobre os objetivos da pesquisa, a formação acadêmica da pesquisadora e o caráter voluntário da participação.

### Local de pesquisa e definição da amostra

A pesquisa foi realizada em quatro Programas de Residência Multiprofissional em Saúde (PRMSs), localizados no interior do RN, vinculados à Universidade Federal do Rio Grande do Norte (UFRN) e à Universidade do Estado do Rio Grande do Norte (UERN), nos municípios de Mossoró, Santa Cruz e Caicó. Inicialmente, foram identificados oito PRMSs no estado, mas optou-se pelos situados fora da capital, a fim de focar na interiorização da formação em saúde.

A amostra foi construída por amostragem teórica, sendo definida ao longo da coleta e da análise de dados, conforme surgiam necessidades de aprofundamento e refinamento das categorias analíticas. Participaram 41 pessoas, divididas em quatro grupos: 30 discentes, seis preceptores, quatro docentes e um gestor de Secretaria Municipal da Saúde. Não foram registradas recusas ou desistências durante o processo de recrutamento e coleta de dados.

A seleção dos participantes foi intencional, considerando a ligação direta com o objeto de estudo. Foram incluídos residentes no último ano, preceptores, docentes e gestores ativos nos PRMSs do interior do RN, com idade mínima de 18 anos. Foram excluídos atores afastados de suas funções, residentes do primeiro ano e aqueles vinculados a residências da capital. A coleta seguiu até atingir a saturação teórica dos dados.

Inicialmente, foram identificados oito PRMSs em todo o território do estado, direcionados a diversas áreas de atuação e vinculados à UFRN e à UERN, com sede em quatro municípios: Natal, Santa Cruz, Caicó e Mossoró. No entanto, vislumbrando a interiorização da formação em saúde na perspectiva da pós-graduação *lato sensu* dos PRMSs, optou-se por trabalhar com os programas situados em cidades do interior do RN. Dessa forma, os quatro PRMSs incluídos na pesquisa são: PRMS em Saúde da Família (UERN/Mossoró); PRMS em Saúde Materno-Infantil (UFRN/Santa Cruz); PRMS em Atenção Primária (UFRN/Caicó); e PRMS em Saúde Materno-Infantil (UFRN/Caicó).

A delimitação dos participantes em pesquisas guiada pela TFD foi realizada mediante a amostragem teórica. Por isso, a amostra do estudo foi sendo construída no decorrer da coleta e análise de dados, conforme as informações foram evidenciadas, demonstrando a necessidade de reunir mais dados para refinar as categorias e elaborar a teoria. Novas informações podem ser necessárias no decorrer do processo e caso a coleta de dados precise ser redirecionada^(7)^.

### Instrumento de coleta de dados

A coleta ocorreu entre novembro de 2022 e agosto de 2023. Inicialmente, realizaram-se GFs com residentes dos PRMSs, técnica que reúne de seis a 12 participantes homogêneos para discutir diferentes perspectivas. Os residentes foram convidados por e-mail, e os encontros ocorreram em ambientes privativos. Planejou-se um GF para cada PRMS, mas realizaram-se apenas dois - um em Mossoró e outro reunindo residentes de Caicó -, atingindo a saturação teórica.

O roteiro dos GFs incluía quatro questões norteadoras sobre a compreensão da educação interprofissional (EIP), a avaliação do processo formativo, o impacto do PRMS nos serviços de saúde e as competências desenvolvidas. Após cada GF, os dados foram transcritos, analisados e diagramados, surgindo novos questionamentos que demandaram ampliação da amostra.

Novas hipóteses levaram à realização de entrevistas semiestruturadas com preceptores, docentes e gestores, também agendadas por e-mail e em locais privativos^(6)^.

No total, foram realizados dois GFs, cada um com duração média de 90 minutos, conduzidos em salas reservadas das instituições parceiras, garantindo privacidade e sigilo. Foram também realizadas 17 entrevistas semiestruturadas individuais, com duração média de 45 a 60 minutos, conduzidas presencialmente em espaços privativos dos serviços de saúde ou instituições acadêmicas. Todas foram gravadas em áudio e transcritas integralmente pela pesquisadora.

Durante a realização das entrevistas e dos GFs, estiveram presentes apenas os participantes e a pesquisadora. A amostra foi composta por residentes, preceptores, docentes e um gestor da área da saúde, com diferentes formações profissionais e tempos de atuação em Programas de Residência Multiprofissional.

Para docentes e preceptores, o roteiro abordou o papel na residência, a organização do trabalho, a avaliação da formação interprofissional, o impacto do PRMS e as competências desenvolvidas. Para gestores, as perguntas focaram no papel da gestão, na articulação com instituições formadoras, nas estratégias de fortalecimento do PRMS e nos impactos no território.

Durante a realização dos GFs e das entrevistas, foram registradas notas de campo, contemplando observações relacionadas ao ambiente de coleta, às interações entre os participantes, às expressões não verbais, aos momentos de concordância ou tensão nos discursos e às impressões analíticas preliminares da pesquisadora. Essas notas foram incorporadas ao processo analítico como material complementar, contribuindo para aprofundar a interpretação dos dados e fortalecer o rigor da análise qualitativa.

Cada entrevista foi transcrita e analisada até a saturação teórica, sem surgimento de novas propriedades ou dimensões nos dados.

Não foram realizadas entrevistas repetidas. As transcrições das entrevistas e dos GFs não foram devolvidas individualmente aos participantes, uma vez que a estratégia metodológica adotada priorizou a validação analítica coletiva do modelo teórico emergente. Em vez de devolver as transcrições individualmente, foi realizado um processo de validação das categorias e do modelo teórico com participantes selecionados, o que permitiu verificar a coerência interpretativa dos resultados e fortalecer a credibilidade dos achados.

Antes do início da coleta de dados, os roteiros utilizados nos GFs e nas entrevistas semiestruturadas passaram por um pré-teste com dois profissionais com experiência em RMSs, que não participaram da amostra final do estudo. Esse procedimento teve como objetivo avaliar a clareza das perguntas, a pertinência dos temas abordados e o tempo médio de aplicação. Após essa etapa, foram realizados pequenos ajustes na redação de algumas perguntas para favorecer maior compreensão e estimular narrativas mais aprofundadas dos participantes.

### Tratamento e análise dos dados

A análise foi realizada de forma simultânea à coleta, permitindo validar os conceitos e as hipóteses emergentes^(8)^. Após a transcrição dos dados, foram elaborados memorandos e diagramas para organização e armazenamento das informações, usados ao longo de toda a pesquisa.

O processo de construção das categorias ocorreu de forma progressiva e iterativa, conforme os princípios da TFD. Inicialmente, durante a codificação aberta, os dados foram fragmentados em unidades de significado, originando códigos iniciais derivados diretamente dos discursos dos participantes. Em seguida, na codificação axial, esses códigos foram comparados, agrupados e reorganizados em categorias e subcategorias, com base em suas propriedades e dimensões. Por fim, na codificação seletiva, realizou-se a integração das categorias em torno de um fenômeno central, permitindo a construção do modelo teórico explicativo do processo formativo nas RMSs.

A análise seguiu três etapas de codificação^(7)^:

- Codificação aberta: identificação de códigos preliminares a partir dos dados brutos, formando categorias e subcategorias iniciais;
- Codificação axial: reagrupamento e integração de categorias e subcategorias, explicando suas propriedades, dimensões e fenômenos relacionados^(8)^;
- Codificação seletiva: construção do esquema teórico explicativo, com identificação da categoria central e formulação de uma teoria substantiva ou modelo conceitual^(9)^.

O modelo paradigmático foi utilizado para estruturar o fenômeno estudado, conectando fenômeno, ações-interações, condições e consequências.

A codificação e a análise de dados foram realizadas pela pesquisadora principal, seguindo as etapas de codificação aberta, axial e seletiva, com rigor analítico assegurado pela comparação constante e pela saturação teórica.

Ao longo desse percurso analítico, foram elaborados memorandos teóricos e diagramas analíticos, que auxiliaram na visualização das relações entre categorias, favorecendo a densidade conceitual e a construção do modelo paradigmático.

A análise não utilizou *softwares* específicos, como NVivo ou Atlas.ti. Optou-se por conduzir a codificação manualmente, seguindo rigorosamente os procedimentos propostos pela TFD straussiana. A escolha baseou-se na compreensão de que o processo manual favorece maior imersão, sensibilidade teórica e proximidade da pesquisadora com os dados, conforme recomendado pelos autores clássicos da TFD.

Para fortalecer a credibilidade dos achados, realizou-se uma validação junto a um grupo de quatro participantes (dois residentes, um preceptor e um docente), que analisaram a coerência das categorias e do modelo teórico. As contribuições foram incorporadas ao processo final de codificação, sem necessidade de ajustes estruturais, confirmando a consistência analítica.

## RESULTADOS

Durante o processo de análise de dados, organizaram-se os mesmos em três componentes do modelo teórico - ações-interações, condições e consequências -, demonstrado no Quadro 1. A partir das relações entre os dados, surgem as categorias e subcategorias, que contêm elementos próprios, os quais descrevem e explicam o fenômeno abordado. Desse modo, a categoria central do fenômeno desse estudo foi intitulada “Compreendendo o processo formativo interprofissional nas Residências Multiprofissionais em Saúde”.

**Quadro 1:** Organização do modelo paradigmático acerca do processo formativo das Residências Multiprofissionais em Saúde, Mossoró, Rio Grande do Norte, Brasil, 2023

	CATEGORIAS	SUBCATEGORIAS
**CATEGORIA CENTRAL**	Compreendendo o processo formativo interprofissional nas Residências Multiprofissionais em Saúde	-
**AÇÕES-INTERAÇÕES**	Influência da preceptoria no processo formativo	- Atuação dos preceptores de núcleo e de campo são instrumentos importantes para consolidar o trabalho interprofissional. - Ausência de preceptores de campo de todas as áreas disciplinares é um entrave no processo formativo. - Há falta de uniformidade no pagamento de bolsas como estímulo à participação das Residências Multiprofissionais em Saúde. - Ausência de processo contínuo de qualificação dos preceptores.
	Estratégias para a consolidação do trabalho interprofissional	- Os atendimentos compartilhados entre todas as disciplinas estimulam ao trabalho em equipe interprofissional. - Articulação entre as diferentes residências, inclusive as médicas, consolida a colaboração interinstitucional. - Ausência de uniformidade nas reuniões sistemáticas de equipe para refinar os processos de trabalho. - Os atendimentos uniprofissionais são necessários ao processo de trabalho disciplinar e à construção de competências. - A desarticulação do Núcleo de Apoio à Saúde da Família comprometeu o processo formativo das Residências Multiprofissionais em Saúde direcionadas à Atenção Primária.
	Necessidades envolvidas no processo formativo das Residências Multiprofissionais em Saúde	- Presença de vários campos de estágio estimula o trabalho em equipe e a construção de diferentes competências. - Corresponsabilização das gestões municipais pela Residências Multiprofissionais em Saúde é necessária para fortalecê-las no território. - Necessidade de fortalecer a compreensão do atendimento centrado no usuário e das necessidades de saúde como eixo condutor das práticas de saúde. - Estimulo para adesão de docentes e preceptores para atuar nas Residências Multiprofissionais em Saúde.
**CONDIÇÕES**	Influência da formação básica (graduação) no processo formativo na residência	- Ausência de experiências e discussões interdisciplinares em nível suficiente na graduação. - Formação fragmentada, disciplinar e guiada pela lógica biologicista. - Dificuldade no desenvolvimento do trabalho em equipe entre as diferentes disciplinas ainda na graduação.
	Organização pedagógica das Residências Multiprofissionais em Saúde	- Formação organizada em dois anos com componentes teóricos e práticos. - Presença de diversidade de equipamentos sociais como campo de estágio. - Presença de princípios da educação interprofissional. - Disposição fragilizada na organização acadêmica e relação entre os atores sociais envolvidos (docentes, preceptores, discentes e gestores). - Dificuldade na articulação e formação dos preceptores.
	Influência da organização dos serviços de saúde na formação	- Processos de trabalho fragmentados, segregados e majoritariamente disciplinar. - Fragilidade no trabalho em equipe. - Ausência de qualificação para os profissionais de saúde nos serviços. - Atuação da preceptoria de campo e núcleo como instrumento-chave para organização do processo de trabalho.
	Atuação da gestão municipal como instituição executora	- Colaboração entre instituição executora e formadora. - Organização financeira para disponibilizar bolsas e estruturar os serviços de saúde. - Necessidade da formação em serviço para a mudança do perfil profissional e perfil epidemiológico do município.
**CONSEQUÊNCIAS**	Formação de competências nas Residências Multiprofissionais em Saúde	- Percebendo a formação de competências comuns, transversais e colaborativas. - Desenvolvimento de uma prática colaborativa. - Desenvolvimento de um trabalho em equipe interprofissional. - Desenvolvimento da centralidade do cuidado, necessidades de saúde e comunicação interpessoal. - Dificuldades na consolidação da educação interprofissional.
	Impacto da Residências Multiprofissionais em Saúde nos territórios	- Presença das residências nos serviços de saúde qualifica a atenção à saúde. - Desenvolvimento de um cuidado mais integral. - Desenvolvimento de novos fluxos e serviços de atenção especializada. - Criação de vínculos com a população, melhorando a qualidade de vida dos mesmos. - Aumento da resolutividade dos serviços de saúde. - Desenvolvimento do refinamento dos processos de trabalho. - Transformação dos indicadores de saúde dos municípios.

O componente “ações-interações” corresponde às respostas expressas aos eventos ou situações problemáticas. Nele, emergiram discursos que embasaram três categorias e suas subcategorias. A primeira, “Influência da preceptoria no processo formativo”, identificou que a ação dos preceptores é essencial e um dos eixos estruturantes desse processo nas residências, pois esses profissionais atuam como articuladores entre a academia e os serviços de saúde, além de guiar os estudantes pelos processos de trabalho estabelecidos nos campos de estágio. Portanto, a escolha dos preceptores deve ser criteriosa, já que devem estar comprometidos e qualificados com o processo formativo, devido à sua influência direta na formação em serviço e na construção de competências e habilidades. Isso é demonstrado no seguinte discurso:

[...] *a preceptoria de campo, que são os preceptores que atuam e ficam responsáveis por aquela equipe em um determinado serviço. A equipe é composta por todos os residentes de todas as categorias. Aqui a gente trabalha com um serviço base e um serviço de apoio, e eles ficam fixos em um serviço. E tem um preceptor de núcleo, que é um preceptor responsável por cada categoria, todos os residentes da mesma profissão, para reuniões semanais, a fim de preencher qualquer lacuna que o residente venha ter ou para ampliar as discussões sobre as atividades.* (Discente 4)

Já na segunda categoria, “Estratégias para a consolidação do trabalho interprofissional, evidenciaram-se, a partir dos discursos, os instrumentos indispensáveis que são desenvolvidos nas residências e auxiliam na consolidação do trabalho interprofissional. Os atendimentos compartilhados entre as diferentes disciplinas nos campos de estágio e a articulação entre as diferentes residências, incluindo as de medicina, são as principais estratégias utilizadas para estimular o trabalho em equipe de maneira interdisciplinar. No entanto, evidenciou-se também a necessidade de fortalecer as competências disciplinares a partir dos processos de trabalho individuais e dos atendimentos uniprofissionais, como forma de embasar a construção de competências colaborativas e a atuação interprofissional. Isso ficou evidente nos seguintes discursos:

*Hoje, a gente faz um movimento de integrar as residências, inclusive as de medicina, ou seja, o componente curricular seminário de aprendizagem interprofissional é o componente da Atenção Básica, mas que elas compõem também a materno infantil e da medicina da família e comunidade* [...] *e aí essa discussão de caso que antes só acontecia em Atenção Básica agora acontece com todos.* (Docente 3)

Por outro lado, afirma-se também que:

*A interprofissionalidade implica em compartilhar objetivos* [...] *é planejar juntos à execução. Não significa dizer que nós anulamos uma atuação uniprofissional; muito pelo contrário, ela é muito necessária. E aí estava existindo uma confusão conceitual, no qual os residentes faziam os atendimentos compartilhados, calçada educativa, grupos operativos, grupos de gestantes, e se negava a fazer uma atividade individualizada, que seja um atendimento de uniprofissional, e isso não pode acontecer.* (Docente 1)

Na terceira categoria, “Necessidades envolvidas no processo formativo das Residências Multiprofissionais em Saúde”, evidenciou-se que a diversidade de campos de estágio em diferentes níveis de complexidade na Rede de Atenção à Saúde enriquece o processo formativo e auxilia na construção de diversas competências. No entanto, é necessário um maior comprometimento da gestão municipal nessa organização, além de maior estímulo aos docentes e preceptores para fazerem parte das residências, visto que existe uma dificuldade para montar essas equipes. Isso influencia diretamente a necessidade de fortalecer a compreensão do atendimento centrado no usuário e das necessidades de saúde como eixo condutor das práticas de saúde, reafirmado pela fala:

*Hoje, nós temos o consultório LGBT, consultório na rua, hospital da mulher* [...] *eles pagam essas horas que não são na UBS, nessas outras localidades, porque, na verdade, não é nem como um plantão; eles teriam que pagar oito horas semanais que é das 48; só consegue pagar 40 que aí eles pagam em plantões.* (Preceptor 5)

Apesar da convergência em torno da relevância da interprofissionalidade como eixo estruturante da formação nas RMSs, os achados evidenciaram variações e tensões importantes entre programas, campos de prática e categorias profissionais. Enquanto alguns participantes relataram experiências formativas marcadas por forte integração entre residentes, preceptores e serviços, outros destacaram fragilidades na articulação interprofissional, especialmente em contextos nos quais predominam processos de trabalho fragmentados ou baixa adesão dos profissionais dos serviços às propostas da residência.

Observaram-se, ainda, percepções divergentes entre categorias profissionais quanto ao equilíbrio entre práticas compartilhadas e atendimentos uniprofissionais, revelando tensões conceituais e operacionais sobre o papel da interprofissionalidade no cotidiano formativo. Essas diferenças refletem não apenas singularidades institucionais e territoriais, mas também trajetórias formativas prévias e condições organizacionais distintas, reforçando a complexidade do fenômeno investigado e a necessidade de compreender a formação interprofissional como um processo heterogêneo, relacional e contextualmente situado, conforme preconizado pelos critérios de rigor analítico do COREQ-EQUATOR.

Já no componente “condições”, são discutidas as razões dadas pelos participantes para o acontecimento, bem como as explicações para os motivos pelos quais respondem de uma determinada maneira a uma ação. Nesse componente, emergiram discursos que embasaram quatro categorias e suas subcategorias. A primeira categoria, “Influência da formação básica (graduação) no processo formativo na residência”, evidencia que a ausência de discussões sobre EIP e de experiências interdisciplinares nas graduações fragiliza o trabalho em equipe e dificulta o processo formativo das residências na perspectiva interprofissional. Isso foi reafirmado pelo discurso:

*Hoje, eu vejo especialmente pelo fato de trabalhar muito junto os três programas de residência. A nossa maior dificuldade é de como o residente chega aqui, porque, principalmente, e não é falando mal, mas algumas graduações não têm discussão do SUS e nem experiência interdisciplinar. Ele é muito acostumado lá na caixinha dele, a só fazer ensino e não trabalhar junto com as outras profissões.* (Docente 2)

Na segunda categoria, “Organização pedagógica das Residências Multiprofissionais em Saúde”, foi exemplificado como essas residências se organizam pedagogicamente em carga horária, módulos e atividades. Salientou-se a existência dos princípios da EIP na estrutura pedagógica dos cursos. No entanto, revelou-se uma grande dificuldade na articulação entre os atores sociais envolvidos em reuniões, projetos e discursos. Isso é demonstrado na fala a seguir:

*É a residência multi. Ela, da forma como ela foi pensada, ela não atende a educação interprofissional. É, no entanto, nós fizemos a opção de ter a educação interprofissional como um dos princípios formativos, né? E aí nós temos um, dois, na verdade, componentes curriculares, mas nós temos um componente curricular que se chama “seminários de aprendizagem interprofissional e trabalho interprofissional”, então nós temos dois componentes curriculares que faz essa discussão.* (Docente 1)

A terceira categoria, “Influência da organização dos serviços de saúde na formação”, evidencia a hegemonia da lógica hospitalocêntrica e biologicista nos serviços de saúde como uma dificuldade para o trabalho em equipe interdisciplinar, pois estimula uma prática profissional fragmentada e disciplinar. Desse modo, salienta-se a necessidade de qualificações constantes dentro dos serviços de saúde, bem como a escolha de preceptores estratégicos para atuarem nesses espaços e agirem como instrumentos de transformação da realidade. Isso será demonstrado a seguir:

*Eu acredito que a diferença da residência é ver realmente na prática, porque a gente ainda ouve falar pouco de interprofissionalidade na graduação; é algo que você só escuta, você não vivencia, eu acho que é um destaque muito grande da gente tá inserido nessa realidade. A grande maioria é pratica; a gente vem descobrir o que é interprofissionalidade dentro do atendimento interprofissional. Mas é muito difícil na prática esse trabalho em equipe. Temos dificuldades com alguns profissionais nos serviços: os mais antigos.* (Discente 10)

Na quarta categoria, “Atuação da gestão municipal como instituição executora”, evidenciou-se que, embora a gestão reconheça a necessidade de formação em serviço para a mudança do perfil profissional e epidemiológico do município, ela ainda atua de maneira discreta e pouco articulada com a instituição formadora, o que fragiliza o processo formativo. Isso foi reafirmado pelo discurso:

*O papel da gestão* [...] *ela é fundamental para apoiar esses movimentos. A gestão, hoje, entende a importância da residência; valoriza e apoia, faz um movimento muito de ajudar a execução das atividades estratégicas da residência, mas ainda precisa se articular melhor com a gente.* (Docente 4)

Por fim, no componente “consequências”, que se refere aos resultados previstos ou reais, emergiram duas categorias e suas subcategorias. Na primeira categoria, “Formação de competências nas Residências Multiprofissionais em Saúde”, percebeu-se que, mesmo com as dificuldades no processo formativo, há, de fato, a formação de competências comuns, transversais e colaborativas entre os estudantes. Constatou-se também que a comunicação interpessoal é fortalecida durante o processo e que é possível desenvolver uma prática colaborativa e um trabalho em equipe interprofissional focado nas necessidades de saúde do sujeito e centrado no paciente. Essa ideia foi reafirmada nos discursos a seguir:

*As competências que a gente trabalha intencionalmente, ou seja, há uma intencionalidade clara; a principal é a centralidade do usuário. É das necessidades de saúde dos usuários, centralidade, o trabalho interprofissional, a comunicação. A gente compreende que a centralidade é quem dá o foco das integrações das articulações interprofissionais, não é em razão disso* [...]. (Docente 2)

*A educação interprofissional não é apenas juntar pessoas, estudantes e diferentes profissionais em um mesmo espaço; é muito mais complexo do que isso; é muito mais que um encontro; requer uma disponibilidade, então não é tão simples como a gente imagina. Não se pode banalizar e, se caso a gente banalize, a gente vai ver lá na frente que nós não vamos enxergar os efeitos da educação interprofissional. O problema não é a educação interprofissional, o problema é que nós nunca fizemos educação interprofissional.* (Docente 3)

Na segunda categoria, “O impacto das Residências Multiprofissionais em Saúde nos territórios”, demonstrou-se que a presença das residências nos serviços de saúde qualifica a assistência, aumenta a resolutividade dos serviços, melhora os indicadores de saúde do município, torna o cuidado mais integral, refina os processos de trabalho, cria novos fluxos assistenciais e melhora a articulação entre os níveis de atenção à saúde. Além disso, impacta a qualidade de vida dos sujeitos assistidos por esses serviços. A questão da localização das RMSs no interior do estado não foi amplamente discutida nem considerada de grande importância nos discursos, o que sugere que o impacto das RMSs independe de sua localização. Isso foi reafirmado pelo discurso:

*Eu, olhando hoje o serviço que eu estou, ainda como um todo, eu acho que nossos impactos, ele é muito mais na vida de comunidade, né* [...] *na comunidade* [...] *as ações que a gente consegue prestar para essa população no momento que eu consigo me reunir com uma equipe interdisciplinar, com um atendimento íntegro* [...] *só é feito nos serviços que tem os residentes, por isso a gente tem um impacto na vida da família, onde a gente realiza o atendimento, então, para mim, isso já é o grande impacto da nossa existência* [...] *levar a qualidade de vida para quem no seu contexto social, no seu contexto econômico jamais conseguiria isso* [...]. (Discente 15)

Para apresentação do modelo teórico, foi construída uma figura ilustrativa acerca do processo formativo interprofissional nas RMSs, exemplificada na Figura 1.

**Figura 1:** Diagrama do modelo teórico acerca da formação interprofissional nas Residências Multiprofissionais em Saúde, Mossoró, Rio Grande do Norte, Brasil, 2023

Nesse modelo, há o construto teórico das categorias que emergiriam no discurso de todos os participantes, sintetizando os principais elementos do fenômeno estudado. Como se percebe, os conceitos discutidos possibilitaram identificar os processos, as práticas e as dificuldades envolvidos no processo formativo interprofissional nas RMSs.

## DISCUSSÃO

Esses resultados convergem com pesquisas que evidenciam o papel central das práticas colaborativas na qualificação do cuidado e no fortalecimento da comunicação entre profissionais. Estudos mostram que ambientes de aprendizagem integrados favorecem competências comuns e colaborativas, essenciais para o trabalho em equipe e para a integralidade da atenção^(10,11)^.

Essa mudança desloca a formação em saúde, que é uniprofissional, biomédica, fragmentada e especializada, para uma abordagem interdisciplinar e interprofissional, ancorada no trabalho em equipe, na integralidade do cuidado e na colaboração entre os profissionais de saúde^(12,13)^.

Além disso, também estimula a consciência social ampliada, atendendo a demandas complexas por meio de práticas inovadoras e colaborativas, e fortalecendo o cuidado integral em saúde centrado no sujeito, a formação e o trabalho em saúde a partir da perspectiva da interprofissionalidade^(14)^. Auxilia no desenvolvimento de competências e habilidades nos discentes, como negociação, mediação, tolerância, dinamicidade, flexibilidade, autonomia, liderança, iniciativa, empatia e dialogo, além de estimular práticas compartilhadas e esclarecer os papéis e a importância de cada categoria profissional na equipe de saúde^(14)^.

Portanto, a formação em serviço, desenvolvida pelas RMSs, cria um espaço privilegiado de atuação interdisciplinar na realidade do trabalho, com potencial para o desenvolvimento de novas tecnologias de cuidado e de práticas pedagógicas, como a EIP, principalmente quando se consideram os aspectos regionais e as necessidades territoriais, direcionando um ensino comprometido com suas responsabilidades sociais e com o desenvolvimento de um trabalho em equipe interprofissional^(10)^.

A EIP é considerada uma estratégia de ensino-aprendizagem comprometida em preparar os trabalhadores para a colaboração interprofissional e o trabalho em equipe. Ela é articulada intersetorialmente para atender à crescente complexidade das necessidades de cuidado da população, focando na integralidade, e consequentemente impacta na melhoria da educação, da dinâmica de trabalho e da saúde^(15)^.

A EIP é caracterizada pela oportunidade de duas ou mais profissões aprenderem juntas, de maneira integrada, desenvolvendo habilidades para o trabalho em equipe e a colaboração profissional com um objetivo em comum. Baseia-se em uma aprendizagem interprofissional, a partir de métodos que estimulam a interação e o compartilhamento de saberes e responsabilidades, oportunizando processos de trabalho em equipe e colaboração entre os profissionais^(10)^.

Apesar desses avanços, persistem fragilidades estruturais, como a pouca articulação entre gestão municipal e instituições formadoras, as dificuldades nos processos de trabalho dos serviços e as limitações decorrentes da formação prévia dos profissionais, frequentemente marcadas pela fragmentação disciplinar. Esses elementos influenciam diretamente a consolidação do trabalho colaborativo e a efetividade das experiências formativas^(6,12)^.

Logo, o ambiente de aprendizagem interprofissional na formação em saúde otimiza a resolução de problemas, facilita o trabalho em equipe, reforça o papel de cada profissional, estimula atitudes positivas no local de trabalho, melhora a comunicação entre os profissionais e qualifica o cuidado ao paciente^(16)^.

Desse modo, a EIP desenvolve um processo de ensino-aprendizagem planejado de maneira interprofissional baseado em um sistema de competências específicas, comuns e colaborativas^(17)^. Essas competências fortalecem a formação de profissionais aptos ao trabalho em equipe interprofissional, respeitando as identidades profissionais e os limites existentes entre elas^(18)^.

Consequentemente, o processo formativo baseado na EIP deve ser direcionado ao aperfeiçoamento de habilidades individuais e coletivas para desenvolver o cuidado centrado no paciente. Esse processo deve ser fundamentado em experiências de aprendizado que oportunizem dinâmicas grupais, discussões de caso, construção de Projetos Terapêuticos Singulares *etc*., visando o fortalecimento da comunicação, da confiança e da atuação em equipe. É essencial que os participantes debatam ideias, definam objetivos centrais e tarefas, tomem decisões de maneira compartilhada, assumam responsabilidades e gerenciem conflitos^(12)^.

Vale salientar a importância do atendimento compartilhado ou interconsulta, reconhecido como uma atividade interdisciplinar entre profissões de diferentes categorias, objetivando um cuidado mais integral, centrado no paciente e com maior resolutividade. No momento desse atendimento, é possível o compartilhamento de saberes interdisciplinares, desenvolvendo uma prática interprofissional^(19)^.

No entanto, os atendimentos individuais e a prática uniprofissional não podem ser negligenciados durante o processo, visto que é necessário construir competências específicas, que são oportunizadas no processo de trabalho individual e disciplinar. Portanto, pensar em interprofissionalidade não é desconsiderar a atuação disciplinar, que também é necessária, mas é vislumbrar que é possível formar competências diferentes em ambos os processos^(20)^.

Além disso, os PRMSs são capazes de transformar os serviços de saúde, visto a integração íntima entre docentes-preceptores-residentes dispostos a refletir criticamente e atuar sobre a realidade^(5)^. Dessa perspectiva, enfatiza-se o impacto positivo do papel do preceptor nos programas de residência. O preceptor é responsável pela supervisão direta das atividades práticas dos residentes, conectando-os ao serviço de saúde e orientando suas práticas, contribuindo, assim, para mudanças coerentes com as políticas públicas e a atenção integral à saúde^(21)^.

No entanto, os preceptores devem ter conhecimentos mínimos relativos ao processo de ensino-aprendizagem, ao currículo, aos objetivos e aos valores do programa de residência, para que, assim, exerçam sua função de maneira adequada, reconheçam o modelo de competência proposto e fortaleçam a integração entre ensino, serviço e comunidade^(21)^.

A gestão municipal também é um dos atores sociais envolvidos no processo formativo das RMSs. Embora os mesmos identifiquem a potencialidade das residências na qualificação dos serviços de saúde e participem do planejamento das ações, principalmente na estruturação da rede de atenção, percebe-se que ainda é em nível insatisfatório. As pesquisas, em sua maioria, demonstram uma escassez de estratégias locais da gestão para o fortalecimento das residências, as quais são incorporadas ao funcionamento cotidiano dos serviços de saúde sem grandes adequações ao processo de ensino-aprendizagem^(22)^.

Por outro lado, a complementação de bolsas para discentes, docentes e preceptores pela gestão municipal, assim como programas de educação permanente e de contratação de egressos das residências, aparece como uma das principais iniciativas de fortalecimento das residências nos territórios. Entretanto, essas não são medidas unânimes previstas no regulamento das RMSs, e por isso nem todos os programas lançam mão dessas estratégias^(22)^.

A potencialidade da RMS é notória e unânime. Para os gestores, a presença das residências amplia o acesso aos serviços, fortalece a produção de vínculos, aumenta a qualidade da atenção e a satisfação do cuidado prestado à população^(22)^. Já os egressos reconhecem o impacto desse processo formativo para sua prática profissional, sentindo-se mais qualificados e eficientes, o que contribui com a resolutividade do sistema de saúde e a qualidade de vida da população^(23)^.

Outro elemento importante é a gestão da clínica desenvolvida pelos residentes, que é pautada na demanda do território, além de considerar a heterogeneidade regional, monitorar o cuidado continuado, estratificar os riscos e organizar as linhas de cuidado. Essa realidade impacta principalmente a medicina, que rotineiramente atua com enfoque curativo, mas que, ao atuar em conjunto com as residências multiprofissionais, tende a ancorar sua prática no paradigma da promoção da saúde com práticas centradas no sujeito, facilitando o trabalho em equipe e a colaboração profissional^(23)^.

Portanto, a presença das RMSs nos territórios é capaz de reestruturar os serviços, implantar novas ações e fluxos assistenciais, mobilizar a educação permanente, qualificar os profissionais e a assistência, impactando positivamente a gestão do cuidado e potencializando as características de intervenção no território. Os achados deste estudo convergem com as perspectivas internacionais da interprofissionalidade descritas por Reeves *et al*. e Barr *et al*., especialmente no que se refere à necessidade de estruturas institucionais que sustentem práticas colaborativas e ambientes de aprendizagem participativos. De modo semelhante, os princípios de centralidade do usuário, comunicação e colaboração, amplamente discutidos no *WHO Framework*, também emergiram como fundamentos estruturantes na formação desenvolvida pelas RMSs investigadas^(24-26)^.

Dessa forma, fortalecer a formação nas RMSs requer estratégias que qualifiquem os preceptores, institucionalizem os espaços coletivos de discussão e consolidem as práticas colaborativas no cotidiano dos serviços. Esses investimentos são fundamentais para sustentar um modelo de formação que responda às necessidades dos territórios e consolide a interprofissionalidade como eixo estruturante da prática em saúde^(10,18)^.

### Limitações do estudo

O presente estudo apresenta como principal limitação a realização em somente um estado federativo, especificamente nas residências interiorizadas. Considera-se que essa delimitação possibilita uma compreensão da realidade local; no entanto, há limitações para a generalização dos resultados para outros contextos, esferas regionais e nacionais. Todavia, surgem também como limitações a ausência de residentes do primeiro ano e a não inclusão de todos os programas existentes no estado, que podem ter inviabilizado uma análise abrangente do estado.

### Contribuições para a área da saúde

O estudo contribui significativamente para a área da saúde pública ao oferecer um modelo teórico que explica o processo formativo interprofissional nas RMSs, considerando a perspectiva dos diferentes atores sociais envolvidos. Dessa forma, ele pode subsidiar ações pedagógicas, estratégias de educação permanente e políticas públicas voltadas à qualificação das residências. Ele viabiliza o fortalecimento da EIP como diretriz formativa essencial, com potencial para transformar práticas profissionais e a organização dos serviços de saúde.

## CONCLUSÕES

Conclui-se que o modelo teórico construído acerca do processo formativo interprofissional nas RMSs é capaz de explicar como esse fenômeno ocorre na prática, considerando a influência de todos os atores sociais no processo formativo, as estratégias necessárias para consolidar a educação e o trabalho interprofissionais, bem como os equipamentos sociais necessários para desenvolver a formação em serviço, vislumbrando também as dificuldades de todo o processo.

Portanto, o modelo teórico orienta a formação interprofissional em saúde, por meio do fortalecimento da EIP e da sensibilização quanto às necessidades de reestruturação do processo pedagógico e estrutural desenvolvidos atualmente nas RMSs. Propõe-se, também, servir de base para a prática interprofissional em saúde.

Espera-se que este estudo contribua para as disciplinas da saúde e para a ciência da enfermagem, minimizando a lacuna de conhecimento relacionada à TFD e qualificando a pesquisa científica e as discussões sobre interprofissionalidade e formação interprofissional em saúde. O estudo apresenta como limitação abordar apenas o estado do RN.

## Data Availability

Os dados de pesquisa estão disponíveis no corpo do artigo.
